# Supervision trajectories of male juvenile offenders: growth mixture modeling on SAVRY risk assessments

**DOI:** 10.1186/s13034-018-0222-7

**Published:** 2018-02-27

**Authors:** Ed L. B. Hilterman, Ilja L. Bongers, Tonia L. Nicholls, Chijs van Nieuwenhuizen

**Affiliations:** 1 GGzE Center for Child & Adolescent Psychiatry, Eindhoven, The Netherlands; 2 Tilburg University, Scientific Center for Care and Welfare (Tranzo), Tilburg, The Netherlands; 3 Justa Mesura, Consultancy & Applied Research, Barcelona, Spain; 4 Department of Psychiatry, University of British Columbia, Vancouver, Canada; 5 BC Mental Health and Substance Use Services, Forensic Psychiatric Services Commission, Coquitlam, Canada

**Keywords:** Dynamic risk factors, Juvenile offenders, Growth mixture modeling, Risk assessment, Risk/need trajectories, SAVRY

## Abstract

**Background:**

Structured risk/need assessment tools are increasingly used to orientate risk reduction strategies with juvenile offenders. The assumption is that the risk/need items on these tools are sufficiently sensitive to measure changes in the individual, family and/or contextual characteristics of juvenile offenders. However, there is very little research demonstrating the capacity of these tools to measure changes in juvenile offenders. Congruent with the developmental and life-course criminology theories (DLC) the objective of this study is to explore the existence of heterogeneous trajectories of juvenile offenders across the juvenile justice system as measured through five empirical risk/need areas based on the Structured Assessment of Violence Risk in Youth (SAVRY), one of the most widely applied risk assessment tools for juveniles.

**Methods:**

This longitudinal study included 5205 male juvenile offenders who transitioned through the Catalan juvenile justice system between 2006 and 2014. During intervention they received at least two, and a maximum of seven, consecutive SAVRY risk/need assessments over an 18-month period. The heterogeneity of latent class trajectories was explored through growth mixture modeling (GMM). The trajectory class membership was linked to covariates through multinomial logistic regression analyses.

**Results:**

Through GMM three to four heterogeneous trajectories, with high quality of separation, were identified in each of the risk/need areas. The trajectories with low risk/needs (45–77% of the sample) remained low and presented a very limited increase in risk/needs during the 18-month period. The high risk/need trajectories (20–37% of the sample) showed a limited decrease or no change. Between 5 and 13% of the sample had large reductions in their risk/needs levels, and approximately 5% showed a large increase in risk/needs.

**Conclusions:**

In line with the DLC theories this study shows that trajectories on criminogenic risk/needs can be heterogeneous and indicate distinct rates of change over time. The results of this study also may suggest a limited sensibility to measure change over time of SAVRY’s risk and protective items. Suggestions to improve the sensitivity of measuring change over time, such as shorter time frames or future-oriented time frames for the scoring of the items, are offered.

## Background

Juvenile offenders have been characterized as ‘moving targets’ [1]. This refers to the short-term behavioural change juvenile offenders can exhibit, and reflects the particular importance of dynamic criminogenic needs for the orientation of risk reduction strategies with adolescents. Especially in violence risk assessment with juvenile offenders these criminogenic needs gained importance in the shift from a prediction-oriented model to a need-oriented model. Validated risk and need assessment tools played an important role in this change [2]. According to the need-oriented model, risk and need assessment tools are used to guide risk reduction strategies that target specific criminogenic needs that are relevant in individual cases [3]. The underlying assumption is that the targeted risk and need factors have the capacity to change and that these variables, as defined on risk assessment measures, are sensitive to change over time.

Adolescent offending trajectories can change under the influence of emotional processes [4], intimate relations, neighbourhood and community processes, and life-events related to family, education or work [5]. As a result of these processes and life-events changes in offending trajectories during adolescence are common [5] and can have distinct implications such as desisting, escalating, and persisting trajectories [6]. Also the involvement in the juvenile justice system can have effects on desistence or persistence in delinquent behaviour. Juvenile justice programs are intended to reduce juvenile delinquency. However, these programs often are unavailable or poor in quality [7] or do not sufficiently address the criminogenic needs of the juveniles [8]. Illustrating these problems, Viljoen et al. found that treatment received by juvenile offenders was not associated with change in their risk/needs [9]. It is important to match treatment to the individual’s risk/needs, after a comprehensive risk/need assessment [10].

The Structured Assessment of Violence Risk in Youth (SAVRY) [11] is one of the most widely used risk and need assessment tools for juvenile offenders. Unfortunately, there is very little known about the extent to which SAVRY is capable of measuring the changes that juvenile offenders experience during adolescence. In a retrospective study in Canada, Viljoen et al. [12] found that approximately one-third part of the sample of 163 juvenile sex offenders, who attended a residential cognitive-behavioural program, showed a decrease in their average scores on the SAVRY dynamic risk items and 8% had an increase on the average scores of the protective items. In a longitudinal study with male (*n* = 107) and female (*n* = 49) juvenile offenders, Viljoen et al. [13] reported that SAVRY reassessments during the probation period were no more predictive than the initial assessment at the start of the probation, suggesting that the average change in risk/needs between the two moments in time had no influence on the predictive validity of the risk assessment. In another Canadian study of 146 juvenile offenders (69.2% male) on probation, Viljoen et al. [9] found that the SAVRY mean-level change scores (baseline scores minus follow-up scores) of the risk and protective items showed limited internal sensitivity to change (i.e., change over time).

These studies were based on the assumption that change trajectories are homogeneous and reflect linear change within the population of interest. However, if change trajectories are not homogeneous but heterogeneous and consist of distinct change trajectories, for example one group with decreasing risk scores and another with increasing scores, the averaging change scores across subgroups could disguise changes, falsely indicating there is no transition over time. The identification of heterogeneous trajectories of change over time is congruent with the developmental and life-course criminology theories (DLC) [14]. According to the DLC theories, the assessment of developmental trajectories of risk and protective factors through longitudinal research can assist in the understanding of within-individual changes over time [15].

Following the DLC theories on the heterogeneity of developmental trajectories, the current study explored the existence of heterogeneous trajectories measured by risk and protective items of male juvenile offenders assessed on SAVRY [11]. The principal research question of this study was: Are there distinct developmental trajectories of male juvenile offenders across the juvenile justice system measured through five empirical risk/need domains based on SAVRY items? Our second question examined whether antecedents (e.g., prior violent and non-violent offenses, previously detained), demographics (e.g., age) and characteristics of the transition through the juvenile justice services could differentiate between any trajectories that emerged.

## Methods

### Sample

The multiyear sample consisted of 5205 male juvenile offenders for whom at least two SAVRY risk assessments were completed by professionals of the Catalonian juvenile justice system, in the period from January 2, 2006 until September 10, 2014. At the time of the first SAVRY assessment the juveniles had an average age of approximately 17 years (*M* = 17.64; *SD* = 1.40). When they were charged for their first offense, their mean age was 15.72 years (*SD* = 1.13). The majority of the sample was of Spanish origin (*n* = 2883, 55.4%); the remainder were of European (*n* = 191, 3.7%), African (*n* = 1065, 20.5%), South American (*n* = 989, 19.0%) or Asian (*n* = 77, 1.5%) origin. The mean total score of the first SAVRY assessment was 16.08 (*SD* = 9.24), with a range from 0 to 45 (theoretical range 0–48). The average total score of the protective factors was 2.71 (*SD* = 1.95), with a range of 0 to 6. Before the first SAVRY assessment, 86.2% (*n* = 4489) of the male juveniles had been charged with at least one violent offense (*M* = 4.51, *SD* = 5.10, *Mdn* = 3.00), and 68.0% (*n* = 3541) with at least one non-violent offense (*M* = 4.05, *SD* = 4.83, *Mdn* = 3.00). The proportion of juveniles with a history of youth detention was 17.3% (*n* = 898, *M* = 2.91, *SD* = 2.64). Measured on the number of imposed sentences (*N* = 13,228), the average duration of community probation in the research period was approximately 12 months (*M* = .97 year, *Mdn* = .74, *SD* = .71), and the average duration of detention (*N* = 7221) in a youth detention centre was .64 years (*Mdn* = .49, *SD* = .69).

Between 2006 and 2014 a total of 79,223 SAVRY assessments were completed for the sample. Due to the different duration of penal interventions the juveniles had been subject to a variable number of consecutive SAVRY assessments during their juvenile justice intervention. At the start of the intervention all juveniles had at least two consecutive SAVRY risk assessments, 85.23% (*n* = 4436) had three consecutive assessments, 66.67% (*n* = 3470) had four assessments, 50.70% (*n* = 2639) had five assessments, 38.89% (*n* = 2024) had six assessments and 30.41% (*n* = 1583) had seven consecutive SAVRY assessments over the 18 month study period. The total of SAVRY assessment included in this study was 24,562.

### Setting

**Fig. 1:**
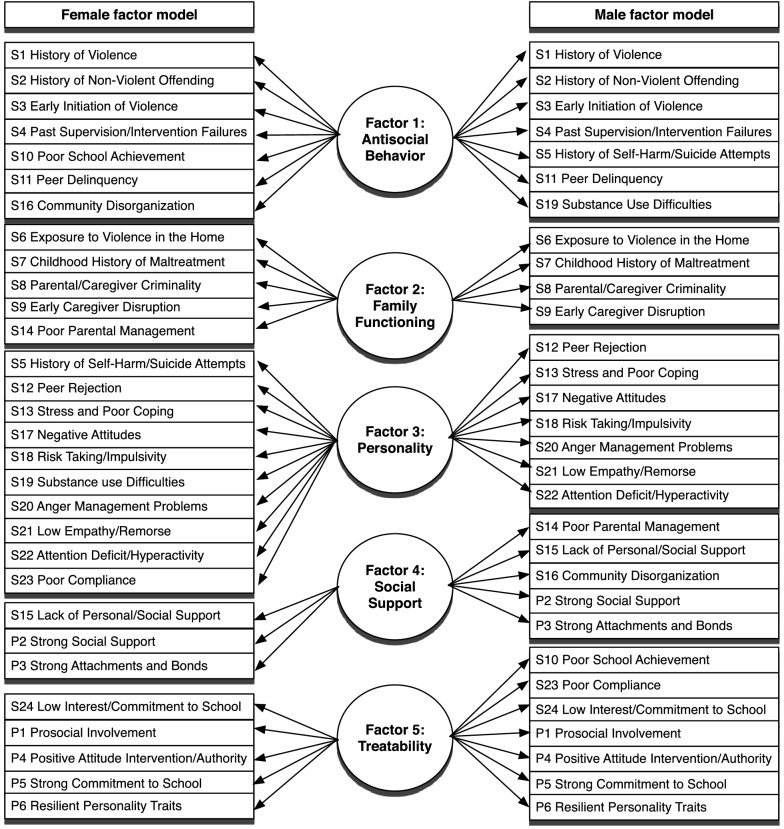
Factor solution for SAVRY risk and protective factors for male and female juvenile offenders

The Catalonian Justice department administers sentences for adults and juveniles in Catalonia, Spain. The juvenile sector is divided into three sections, pre-trial assessment, probation, and custody. The pre-trial assessment section is divided into eight units that work in close collaboration with the office of the public prosecutor. The probation section, with nine juvenile probation units, is responsible for the administration of all community sanctions. *Llibertat vigilada*, or community probation, is the most frequently imposed sanction. The custody section consists of six youth detention centres. Juveniles from the different settings of the juvenile system are not necessarily distinct groups. For instance, after detention in a custodial setting, juveniles always have community probation and juveniles who violate the conditions of the probation can be transferred to a custodial setting. The intervention most often applied during the research period was probation supervision, 10.7% of juveniles were also sentenced to detention. Juvenile justice professionals could apply treatment components like group or individual programs directed towards violent or sexual offenders, mental health problems, drug abuse, and/or family problems (Fig. 1).

### Measures

*Structured Assessment of Violence Risk in Youth* (SAVRY) [11, 16] is a risk assessment tool in the structured professional judgment (SPJ) tradition. The SAVRY contains 24 risk items and 6 protective items. Each risk item is scored on a 3-point scale; low (0), moderate (1) or high risk (2). Protective items are scored present (1) or absent (0) (note—for interpretation purposes protective items have been reverse scored). The final summary risk rating is the product of a clinical reflection on the basis of the information gathered on the particular juvenile and is not based on a sum score. For research purposes, the risk and protective items can be summed up into total scores with a range from 0 to 48, and 0 to 6, respectively.

Results regarding inter-rater reliability (intraclass correlation coefficients (ICC), two way random effects model for absolute agreement and single raters) with SAVRY in the Catalonian juvenile justice system have been published elsewhere and ranged from .93 for the historical items to .57 for the protective items [17]. Intraclass correlations are often interpreted as follows: < .40 = poor; .40 to .59 = fair; .60 to .74 = good; and .75 to 1.00 = excellent [18].

Instead of relying on the conceptual subscales of SAVRY proposed by the authors for the current study we used a gender sensitive order of the SAVRY items as per their categorization into five factors representing specific risk/need areas based on prior research (see Fig. 1) [17]. Three fit indices were used to assess the fit of the factor models: the comparative fit index (CFI), the Tucker–Lewis index (TLI) and a root mean square of approximation (RMSEA), CFI and TLI exceeding .90, indicate acceptable fit, while values close to .95 are recommended [19]. A RMSEA value close to .06 indicates a good fit [19]. In exploratory factor analyses performed in Mplus [20], both models had a good fit (Males: CFI = .96; TLI = .94; RMSEA = .053, 90% CI .051, .055); females: (CFI = .95; TLI = .94; RMSEA = .049, 90% CI [.044, .054]) these were replicated in confirmatory factor analyses with the validation sample (Males: CFI = .94, TLI = .93, RMSEA = .059, 90% CI [.058, .061], females: CFI = .91, TLI = .90, RMSEA = .063, 90% CI [.058, .068]). The five risk/needs areas had good internal consistency for both genders (between .81 and .71 for males and .82–.70 for females). For the growth mixture modeling the SAVRY items of each of the five risk/need areas were summed up and divided through the theoretical range of the scale, which resulted in a score between 0 and 1. The SAVRY assessments were obtained from a database that is part of the electronic information system of the Catalonian Juvenile Justice system (*Sistema d’Informació de Justícia Juvenil*, SIJJ). All data were anonymized to avoid identification of participants.

Criminal history data and the below listed covariates were collected for each individual from the information systems for juvenile and adult offenders of the Catalonian Department of Justice. These official databases include records of impending prosecutions, convictions, and the process of the execution of sanctions.

*Age at first assessment* reflects the date of the first SAVRY assessment.

*Prior offenses* were classified as violent versus non-violent as defined in the SAVRY manual and were measured by the number of criminal charges registered before the first SAVRY assessment. Because *prior violent offenses and non*-*violent offenses* were Poisson distributed both variables were recoded into four categories: 0 = zero prior offenses, 1 = first offenders with only one offense preceding the first SAVRY assessment, 2 = 2–9 offenses and 3 = 10 or more prior offenses. The first category, zero prior offenses, was used as the reference category for both variables.

*Previous detention* was measured as a dichotomous variable, with “0” not previously detained, and “1” having been detained before the first SAVRY risk assessment.

*Number of sector changes* An increase in the *number of sector changes* between the three sectors of the juvenile justice system is an indication of a problematic passing through the system. Sector changes were measured from the start of the transition through the juvenile justice system, up until the last SAVRY risk/need assessment that was performed. The minimum value was “0”, and the maximum value was “3”.

*Sentence duration* During the research period the average duration of community probation, the most frequently imposed sanction, was approximately 12 months (*M* = .97 year, *Mdn* = .74, *SD* = .71). The length of the current sentence (*sentence* > *1*-*year)* was measured as a dichotomized variable with the 1-year average duration as reference (1 = *sentence* > *1*-*year*, 0 = *sentence* ≤ *1*-*year*).

### Procedure

According to the protocols that were developed for the implementation of structured risk/need assessments in the Catalonian juvenile justice system, professionals have been using the SAVRY since 2006 for routine risk/need assessments at different points before and during the penal sanction: (a) during pre-trial assessment; (b) at the start of a youth’s probation (*llibertat vigilada*) or detention; (c) every 3 months during the probation period or the youth’s detention; and (d) at the end of the sentence when the last assessment is performed. Following the method of structured professional judgment, SAVRY risk/need assessment was used to identify relevant goals for the intervention with every individual. From 2006 until 2008 the SAVRY was gradually implemented in the whole Catalonian juvenile justice sector. Before using SAVRY, all professionals (i.e., youth probation officers, group leaders, social workers and psychologists) received 20 h of risk assessment training. After the training, individual supervision was provided. Approximately 1–2 years after the initial training professionals participated in an advanced training to refresh practical and theoretical knowledge. Professionals also received ongoing training on how to target criminogenic needs during intervention.

### Statistical analysis

Growth mixture modeling (GMM) in Mplus 7.1 [20] was used to examine the existence of trajectory heterogeneity in the risk and need developmental pathways of juvenile offenders during their intervention in the juvenile justice system. For each possible class GMM estimates the level of the initial status (intercept), linear change over time (linear slope) and nonlinear change over time (quadratic slope) and for each variable the variance and covariance’s were estimated. A slope that is significantly different from zero indicates change over time. Change has a linear form when the trajectory is constant across the time points (linear slope) or a nonlinear curvature form (quadratic slope) when the slope describes accelerating or decelerating trajectories.

The number of latent trajectory classes was estimated using three indicators: the Bayesian Information Criterion (BIC), the Lo-Mendell-Rubin adjusted likelihood ratio test and the class size. A model with a lower BIC value performs better in terms of taking into account both fit and parsimony. The BIC was preferred over the adjusted BIC because of its better performance with continuous outcomes [21]. The Lo-Mendell-Rubin adjusted likelihood ratio test (LMR-LRT) compares the fit of the model with one more class to the previously estimated model with one less class. A significant *p* value of the LMR-LRT indicates that the model with one less class is rejected in favor of the model with one more class. To avoid small classes, models with a class size smaller than 3.0% were rejected as larger classes are preferred to smaller classes which can be less meaningful [21].

The entropy and the average posterior latent class probabilities were used to assess the quality of the trajectory classifications. Both indices have a range between 0 and 1; higher values indicate a better separation between the classes. Entropy values of .80, .60 and .40 indicate high, medium and low class separation [22]. The average latent class probability for each class reflects the highest probability of each individual assigned to a particular class. To avoid incorrect class solutions due to ‘local maxima’ all models were replicated twice using the seed values from the highest log-likelihood values as described by Asparouhov and Muthen [23]. Through these replications it was confirmed that the models were stable and the model parameters were based on the global solution. Adolescents with shorter sanctions have fewer SAVRY assessments and consequently fewer data points. Elimination of these juveniles from the research would have resulted in the absence of juveniles with shorter sanctions. To include these juveniles with less data points in the analysis, the full-information maximum likelihood (FIML) was used to estimate these missing data points of juveniles whose penal sanctions were shorter than 18 months [24, 25].

The trajectory class membership of the five risk/needs areas was linked to covariates through multinomial logistic regression analyses. To eliminate non-significant variables from the equation the backward stepwise method, with the lowest risk/need trajectory classes as reference categories, was used. For the interpretation of the odds ratio (OR) we followed the criteria suggested by previous research [26] in which an OR with a value between 2 and 3 was considered small, 3–5 medium and above 5 large.

## Results

### Selection of the latent trajectory classes

For the risk/need areas antisocial behaviour, family functioning and personality the linear model with intercept and linear slope was sufficient to describe the model, the incorporation of the quadratic slope in the model resulted in LMR-LRT values that were not significant. The models of the risk/need areas social support and treatability improved after the incorporation of the quadratic slope into the model. Because of the better fit of the model, for the risk/need areas social support and treatability we reported the model with the incorporation of the quadratic slope, for the remaining risk/need areas the model with the linear slope was reported. For each of the risk/need areas, the optimal number of latent class trajectories was estimated; fit statistics for the five models are summarized in Table 1.

**Table 1:** Model fit indices for growth mixture models

Model	BIC	LMR-LRT	Entropy	Smallest class %/(*n*)
Antisocial behaviour				
1	− 42,552.08	n/a	n/a	n/a
2	− 43,352.39	*p* < .001	.91	.039 (202)
*3*	*−* *44,042.78*	*p* *<* *.001*	*.80*	*.040 (207)*
4	− 44,209.88	*p* = .006	.84	.005 (28)
5	− 44,286.48	*p* = .33	.83	.006 (29)
Family functioning				
1	− 50,973.55	n/a	n/a	n/a
2	− 52,575.68	*p* < .001	.87	.209 (1088)
*3*	*−* *53,646.03*	*p* *<* *.001*	*.89*	*.033 (170)*
4	− 54,171.66	*p* = .16	.87	.027 (140)
5	− 54,506.86	*p* = .20	.90	.021 (111)
Personality				
1	− 29,962.82	n/a	n/a	n/a
2	− 30,254.27	*p* < .001	.61	.318 (1653)
3	− 30,519.34	*p* < .001	.74	.157 (818)
*4*	*−* *30,721.70*	*p* *<* *.001*	*.69*	*.043 (222)*
5	− 30,775.80	*p* = .081	.70	.015 (78)
Social support				
1	− 24,159.37	n/a	n/a	n/a
2	− 24,714.14	*p* < .001	.72	.337 (1755)
3	− 25,136.04	*p* < .001	.80	.029 (151)
*4*	*−* *25,733.02*	*p* *<* *.001*	*.82*	*.053 (278)*
5	− 25,913.54	*p* = .240	.82	.021 (111)
Treatability				
1	− 18,466.19	n/a	n/a	n/a
2	− 18,845.24	*p* < .001	.65	.416 (2165)
3	− 18,996.01	*p* < .001	.67	.090 (468)
*4*	*−* *19,470.22*	*p* *<* *.001*	*.75*	*.049 (256)*
5	− 19,559.41	*p* = .240	.76	.022 (114)

Entropy and LMR-LRT are not applicable for the 1-class model. The values in italics indicate the selected model

*BIC* Bayesian Information Criterion, *LMR-LRT* Lo-Mendell-Rubin adjusted likelihood ratio test, *Entropy* average quality of classification

For the risk/need area antisocial behaviour the model with three classes was preferred. Although the BIC continued to decrease and the LMR-LRT was significant for the models with four classes, the four-class model identified a class that was too small (.5%, *n* = 28). The entropy (.80) and average posterior probabilities, .93, .87 and .88, for all three classes indicated a clear separation between the trajectories. For the risk/need area family functioning a three-class model fit the data best, a model with four classes was rejected due to the non-significant LMR-LRT (*p* = .16). The classification indices of the three-class model of family functioning indicated a high quality of separation between classes with an entropy of .89 and average posterior probabilities of .97, .89 and .94, respectively. For the personality risk/need area a model with five classes was rejected by the LMR-LRT in favour of a four-class model, moreover the five-class model had one class that was too small (1.5%, *n* = 78). The entropy of the four-class model was .69, and the average posterior probabilities were .76, 80, .87 and .80 for the classes one to four, indicating a slightly above medium quality of class separation. For the risk/need areas social support and treatability also the quadratic slope was included in the model, this resulted in improved class separation and indicated nonlinear development over time. The four-class model fit the data best for the social support risk/need area; the LMT-LRT rejected the five-class model, which also had one too small class (2.1%, *n* = 111). For treatability, the last risk/need area based on SAVRY, a four-class model was also preferred. Although the BIC value of the five-class model was smaller compared to the BIC of the previous model, the difference was small, as was the difference between the entropy values (5-class model = .76, 4-class model = .75). The five-class model contained one class which was under the 3.0% cut-off value for the smallest class (2.2%, *n* = 114). The average posterior probabilities of the four-class model were, .86, .88, .84, and .84, respectively.

### The developmental processes of the risk/needs areas

**Fig. 2:**
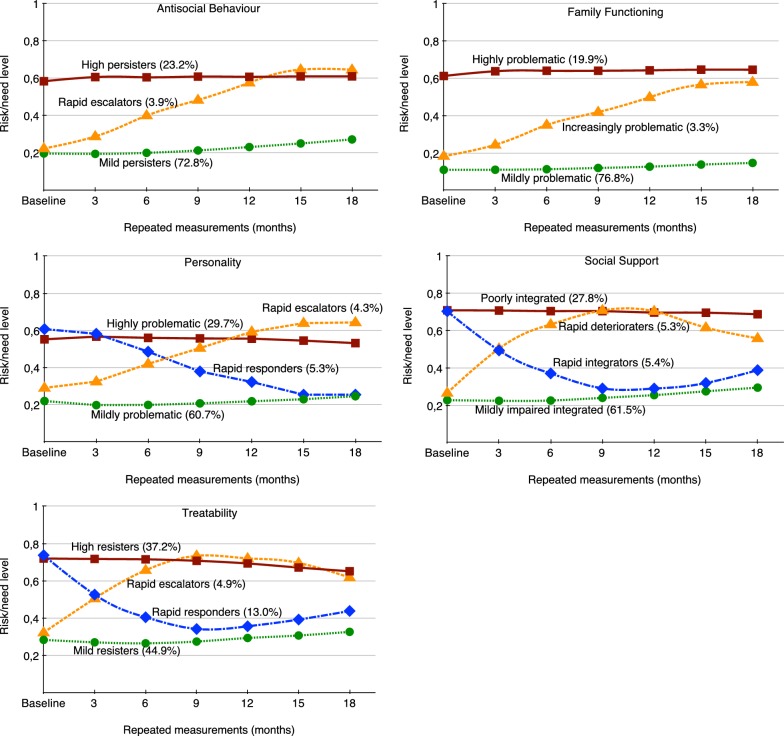
Latent class trajectories of the five risk/need areas based on SAVRY risk and protective factors

The latent class trajectories of the different risk/need areas are shown in Fig. 2.

#### Antisocial behaviour

The largest class (mild persisters, 72.8%, *n* = 3788) was characterized by a low initial status of antisocial behaviour (*β*_intercept_ = .195, *p* < .001), which slowly increased during the transition through juvenile justice (*β*_linear slope_ = .002, *p* < .001; see Fig. 2). The second largest class (High persisters, 23.2%, *n* = 1210) had a high initial status of antisocial behaviour (*β*_intercept_ = .604, *p* < .001) and did not change during the study period (*β*_linear slope_ = − .001, *p* > .05). The smallest group, the rapid escalators (4.0%, *n* = 207) had a low initial status of antisocial behaviour (*β*_intercept_ = .214, *p* < .001), and escalated rapidly to a high level (*β*_linear slope_ = .093, *p* < .001).

#### Family functioning

The largest group (76.8%, *n* = 4000) was mildly problematic at the starting point (*β*_intercept_ = .109, *p* < .001), and during consecutive assessments slightly more problems were detected (*β*_linear slope_ = .001, *p* < .01). The second largest class (19.9%, *n* = 1035) was observed to be highly problematic at the initial assessment (*β*_intercept_ = .632, *p* < .001), and this did not change over time (*β*_slope_ = .002, *p* > .05). The smallest group (3.3%, *n* = 170) was increasingly problematic; the initial observation was mildly problematic (*β*_intercept_ = .177, *p* < .001), but during subsequent assessments significant more problems were observed (*β*_linear slope_ = .085, *p* < .001).

#### Personality

The largest class (60.7%, *n* = 3162) was initially characterized by mildly problematic personality traits (*β*_intercept_ = .206, *p* < .001), which changed minimally over the 18-months period (*β*_linear slope_ = − .003, *p* < .01). The highly problematic class trajectory had a high starting point (29.7%, *n* = 1546, *β*_intercept_ = .596, *p* < .001) and exhibited limited improvement during successive assessments (*β*_linear slope_ = − .006, *p* < .001). The rapid responders (5.3%, *n* = 275), had a high initial status (*β*_intercept_ = .648, *p* < .001), but improved rapidly during the intervention (*β*_linear slope_ = − .087, *p* < .001). The rapid escalators (4.3%, *n* = 222) started the trajectory as a mildly problematic group (*β*_intercept_ = .269, *p* < .001), but rapidly became more problematic (*β*_linear slope_ = .076, *p* < .001).

#### Social support

The mildly impaired integrators were the largest group (61.5%, *n* = 3200, *β*_intercept_ = .227, *p* < .001); this group remained stable during the first 6 months of the trajectory (*β*_linear slope_ = − .005, *p* > .05), and deteriorated slowly during the final 12 months (*β*_quadratic slope_ = .002, *p* < .001). Almost a third of the sample (27.8%, *n* = 1448) was poorly integrated at baseline (*β*_intercept_ = .709, *p* < .001), and did not change during subsequent assessments (*β*_linear slope_ = .000, *p* > .05, *β*_quadratic slope_ = − .001, *p* > .05). The rapid integrators had a poor initial status (5.4%, *n* = 279, *β*_intercept_ = .697, *p* < .001), improved rapidly during the first 12 months (*β*_linear slope_ = − .220, *p* < .001) but experienced a limited decrease towards the end of the 18-month period (*β*_quadratic slope_ = .027, *p* < .001). To the contrary, the rapid deterioraters started out as relatively well socially integrated (5.3%, *n* = 278, *β*_intercept_ = .275, *p* < .001), deteriorated rapidly during the initial 9 months (*β*_linear slope_ = .254, *p* < .001), but then improved slightly towards the end of the trajectory (*β*_quadratic slope_ = − .036, *p* < .001).

#### Treatability

The largest group, the mild resisters (44.9%, *n* = 2337), exhibited low levels of treatment resistance at baseline (*β*_intercept_ = .284, *p* < .001), and increased slightly after an initial decrease (*β*_linear slope_ = − .016, *p* < .001, *β*_quadratic slope_ = .003, *p* < .001). The high resisters (37.2%, *n* = 1936, *β*_intercept_, = .719, *p* < .001) exhibited a high degree of resistance initially but improved somewhat after a short stable period (*β*_linear slope_ = .003, *p* > .05, *β*_quadratic slope_ = − .003, *p* < .001). The rapid responders (13.0%, *n* = 676) started with a high degree of resistance initially (*β*_intercept_ = .730, *p* < .001), improved rapidly (*β*_linear slope_ = − .221, *p* < .001), but had a setback after the first 12 months (*β*_quadratic slope_ = .029, *p* < .001). The rapid escalators (4.9%, *n* = 256) started with low-moderate resistance (*β*_intercept_ = .320, *p* < .001), escalated rapidly to high resistance (*β*_linear slope_ = .227, *p* < .001), and improved somewhat in the last part of the intervention (*β*_quadratic slope_ = − .030, *p* < .001), but never returned to anywhere near baseline.

### Association between covariates and latent trajectory class membership

Tables 2 and 3 show the odds ratios and the 95% confidence intervals of the multinomial logistic regression analyses that link the covariates to the latent trajectory class membership of the risk/need areas antisocial behaviour and family functioning (Table 2) and personality, social support and treatability (Table 3). Here we will mainly focus our discussion on medium and large odds ratios.

**Table 2:** Multivariate odds ratios and confidence intervals for predictors of latent trajectory membership for the risk/need areas antisocial behaviour and family functioning (*N* = 5205)

Antisocial behaviour	Latent class trajectories Odds ratios [confidence intervals]	
	Rapid escalators	High persisters
Nagelkerke R^2^ .299		
Age at first assessment	.68** [.61, .77]	.99 [.94, 1.05]
Prior violent offenses		
0 (reference category)	1.00	1.00
1	1.12 [.57, 2.61]	1.46* [1.04, 2.05]
2–9	1.77 [.92, 3.40]	3.02** [2.30, 3.98]
≥ 10	4.67** [2.17, 10.05]	9.10** [6.38, 12.99]
Prior non-violent offenses		
0 (reference category)	1.00	1.00
1	1.83* [1.19, 2.82]	1.96** [1.55, 2.47]
2–9	2.18** [1.53, 3.11]	3.18** [2.64, 3.83]
≥ 10	1.11 [.42, 2.92]	8.46** [6.14, 11.66]
Previous detention	1.47 [.84, 2.59]	2.40** [1.83, 3.14]
Number of sector changes	2.52** [2.16, 2.96]	1.49** [1.36, 1.64]
Sentence > 1 year	2.20** [1.46, 3.32]	2.24** [1.88, 2.66]

Family functioning	Latent class trajectories Odds ratios [confidence intervals]	
	Increasingly problematic	Highly problematic
Nagelkerke R^2^ .067		
Age at first assessment	.83** [.74, .94]	.97 [.92, 1.02]
Prior violent offenses		
0 (reference category)	1.00	1.00
1	2.43* [1.02, 5.76]	.91 [.69, 1.21]
2–9	2.47* [1.12, 5.42]	1.21 [.96, 1.52]
≥ 10	5.35** [2.24, 12.74]	1.98** [1.44, 2.71]
Prior non-violent offenses		
0 (reference category)	1.00	1.00
1	.98 [.59, 1.63]	1.19 [.96, 1.47]
2–9	1.44 [.99, 2.10]	1.41** [1.18, 1.67]
≥ 10	1.87 [.99, 3.55]	2.19** [1.62, 2.96]
Previous detention	1.24 [.73, 2.11]	1.50** [1.16, 1.94]
Number of sector changes	1.55** [1.31–1.85]	1.11* [1.01, 1.22]
Sentence > 1 year	1.75** [1.16, 2.63]	1.36** [1.16, 1.59]

The “mild persisters” trajectory class serves as reference category in the Antisocial behaviour risk/need area, and the “mildly problematic” is the reference category in the Family functioning risk/need area. Previously detained = 1, not previously detained = 0. Duration sentence ≤ 1 year = 0, duration sentence > 1 year = 1

Multinomial logistic regression analyses, with backwards stepwise elimination, was used to link covariates to trajectory class membership

* *p* < .05, ** *p* < .01

**Table 3:** Multivariate odds ratios and confidence intervals for predictors of latent trajectory membership for the risk/need areas personality, social support and treatability (*N* = 5205)

Personality	Latent class trajectories Odds ratios [confidence intervals]		
	Rapid responders	Rapid escalators	Highly problematic
Nagelkerke R^2^ .158			
Age at first assessment	.85** [.78–.94]	.85** [.76–.95]	.87** [.83, .91]
Prior violent offenses			
0 (reference category)	1.00	1.00	1.00
1	1.99* [1.02, 3.86]	1.83 [.93, 3.62]	1.31* [1.01, 1.69]
2–9	2.69** [1.49, 4.86]	1.93* [1.06, 3.52]	1.68** [1.36, 2.09]
≥ 10	5.84** [2.91, 11.73]	5.04** [2.49, 10.21]	4.00** [2.92, 5.50]
Prior non-violent offenses			
0 (reference category)	1.00	1.00	1.00
1	1.14 [.79, 1.64]	1.40 [.90, 2.16]	1.39** [1.15, 1.68]
2–9	1.11 [.82, 1.49]	1.78** [1.26, 2.52]	1.56** [1.33, 1.82]
≥ 10	1.53 [.88, 2.68]	2.35** [1.29, 4.27]	2.18** [1.61, 2.95]
Previous detention	1.62* [1.03, 2.56]	1.12 [.65, 1.92]	1.77** [1.36, 2.30]
Number of sector changes	1.63** [1.40, 1.90]	2.21** [1.89, 2.58]	1.31** [1.20, 1.44]
Sentence > 1 year	1.97** [1.45, 2.69]	2.21** [1.89, 2.58]	1.66** [1.44, 1.91]

Social support	Latent class trajectories Odds ratios [confidence intervals]		
	Rapid integrators	Rapid deterioraters	Poorly integrated
Nagelkerke R^2^ .137			
Age at first assessment	.89* [.81, .98]	.86** [.78, .95]	.96 [.91, 1.00]
Prior violent offenses			
0 (reference category)	1.00	1.00	1.00
1	1.70* [1.00, 2.89]	1.28 [.71, 2.28]	1.13* [.88, 1.46]
2–9	1.61* [1.01, 2.57]	1.62 [.99, 2.66]	1.28* [1.04, 1.58]
≥ 10	1,47 [.75, 2.89]	2.61** [1.39, 4.89]	2.43** [1.79, 3.30]
Prior non-violent offenses			
0 (reference category)	1.00	1.00	1.00
1	1.31 [.91, 1.90]	1.68** [1.17, 2.40]	1.52** [1.24, 1.85]
2–9	1.62** [1.20, 2.19]	1.52** [1.12, 2.06]	2.01** [1.71, 2.36]
≥ 10	2.20** [1.22, 3.97]	1.90* [1.03, 3.49]	3.63** [2.68, 4.91]
Previous detention	2.03** [1.28, 3.23]	1.73* [1.09, 2.76]	1.88** [1.44, 2.46]
Number of sector changes	1.77** [1.51, 2.06]	2.07** [1.79, 2.40]	1.31** [1.19, 1.43]
Sentence > 1 year	1.12 [.85, 1.49]	1.18 [.88, 1.58]	1.80** [1.55, 2.09]

Treatability	Latent class trajectories Odds ratios [confidence intervals]		
	Rapid responders	Rapid escalators	High resisters
Nagelkerke R^2^ .133			
Age at first assessment	.86** [.80, .92]	.84** [.74, .90]	.86** [.82, .90]
Prior violent offenses			
0 (reference category)	1.00	1.00	1.00
1	1.47* [1.00, 2.16]	1.31 [.79, 2.19]	.88 [.70, 1.12]
2–9	2.15** [1.54, 2.99]	1.15 [.73, 1.80]	1.09 [.89, 1.32]
≥ 10	2.07** [1.30, 3.31]	2.03* [1.12, 3.68]	1.27 [.93, 1.74]
Prior non-violent offenses			
0 (reference category)	1.00	1.00	1.00
1	1.43** [1.10, 1.86]	2.01** [1.36, 2.98]	1.71** [1.41, 2.06]
2–9	1.86** [1.50, 2.30]	2.32** [1.66, 3.23]	2.12** [1.81, 2.48]
≥ 10	2.66** [1.73, 4.11]	2.53** [1.32, 4.82]	3.13** [2.26, 4.35]
Previous detention	2.26** [1.57, 3.24]	1.50 [.87, 2.58]	1.88** [1.40, 2.53]
Number of sector changes	1.88** [1.66, 2.12]	1.89** [1.60, 2.24]	1.64** [1.48, 1.80]
Sentence > 1 year	.91 [.75, 1.10]	1.36* [1.00, 1.84]	1.48** [1.29, 1.69]

The “mildly problematic” trajectory class serves as reference category in the personality risk/need area, the “mildly impaired integrators” is the reference category in the Social support risk/need area, and the “mild resisters” is the reference category in the treatability risk/need area

Previously detained = 1, not previously detained = 0. Duration sentence ≤ 1 year = 0, duration sentence > 1 year = 1

Multinomial logistic regression analyses, with backwards stepwise elimination, was used to link covariates to trajectory class membership

* *p* < .05, ** *p* < .01

#### Antisocial behaviour

All variables in the model were significantly associated with trajectory class membership, which resulted in a significant fit (χ^2^(20) = 1326.64, *p* < .001, Nagelkerke *R*^2^ = .299). Under the influence of the other variables, being younger in age increased the odds (OR = .068) of being in the rapid escalator trajectory more than in de mild persisters trajectory (for example, compared to a 17 year old juvenile, a 14 year old had a 4.41 higher likelihood (4.41 = 1/.68 multiplied by the 3 years difference in age) to be in the rapid escalator class). Juveniles who previously committed ten or more violent and non-violent offenses had a higher likelihood (OR = 9.10 for prior violence, OR = 8.46 for non-violent offenses) to be in the high persistent trajectory compared to juveniles with no offenses in the mild persistent trajectory. Adolescents who had a lower frequency of prior violent and non-violent offenses (two to nine) this likelihood was approximately three times higher for each of the offense types. For the rapid escalators only ten or more prior violent offenses increased the likelihood (OR = 4.67) of being in this rapid ascending trajectory, compared to the mild persisters who did not commit violent offenses. Also an increasing number of changes between sectors of the juvenile justice system, an indication of difficulties during the execution of the sanction, increased the odds of being in the rapid escalator trajectory (OR = 2.52 per change). The rapid escalators had a higher average number of changes between the three sectors of the juvenile justice system (*M* = 1.43, *SD* = .88) compared to the mild persisters (*M* = .44, *SD* = .67) and the high persisters (*M* = .81, *SD* = .90, *F*(2, 5202) = 263.65, *p* < .001).

#### Family functioning

All covariates had a significant association with trajectory membership and the fit of the model was significant (χ^2^(20) = 258.45, *p* < .001, Nagelkerke *R*^2^ = .067). However, the effect sizes of the odds ratios were generally small. Juveniles who were previously charged with ten or more violent offenses had a high likelihood (OR = 5.35) of being in the increasingly problematic trajectory compared to juveniles with no violent offenses in the mildly problematic trajectory. Compared to the mildly problematic group, also the number of changes between sectors of the juvenile justice system increased the likelihood of being a member of the increasingly problematic class (OR = 1.55 per change), more so than of being in the highly problematic class (OR = 1.11 per change), although both were significant.

#### Personality

The model had a good fit (χ^2^(30) = 752.41, *p* < .001, Nagelkerke *R*^2^ = .158). Although all covariates had a significant association with trajectory membership, the effect sizes of the odds ratios were generally small. Exceptions were the large to medium odds ratios regarding ten or more prior charges for violent offenses and the trajectory membership of the rapid responders, the rapid escalators and the highly problematic trajectories, which were all significantly different from the mildly problematic trajectory. In additional multinomial logistic analyses, with the highly problematic class as the reference group and the mildly problematic class excluded, only the number of sector changes remained significant (χ^2^(2) = 61.34, *p* < .001, Nagelkerke *R*^2^ = .04, OR = 1.30; CI 95% [1.13, 1.49], for rapid responders, OR = 1.75; 95% CI [1.52, 2.03], for rapid escalators).

#### Social support

The model had a significant fit (χ^2^(30) = 650.19, *p* < .001, Nagelkerke *R*^2^ = .137) and all covariates associated significantly with trajectory membership. The effect sizes of the odds ratios were small; the only odds ratio with a medium effect size was the dummy variable ten or more prior non-violent offenses, which increased the likelihood of being in the poorly integrated trajectory, compared to juveniles with no non-violent offenses in the mildly impaired integrators trajectory. In comparison to the reference group, shifting between sectors of the juvenile justice system was a risk factor for the rapid deterioraters trajectory (OR = 2.07 per change) and rapid integrators trajectory (OR = 1.77 per change) and to a lesser degree for the poorly integrated trajectory (OR = 1.31 per change).

#### Treatability

All variables of the model were associated with trajectory class membership and contributed to the fit of the model (χ^2^(30) = 659.80, *p* < .001, Nagelkerke *R*^2^ = .133). Nonetheless, the effect sizes of the odds ratios, albeit significant, were generally small. In comparison to the mild resister subgroup, the high resisters had a moderate higher likelihood to have been charged previously for non-violent offenses (OR = 3.13). Compared to the mild resisters, the number of sector changes in the juvenile justice system increased the likelihood with 1.88 and 1.89, for every change to be in the rapid escalator or rapid responder trajectories, for the high resister trajectory this likelihood was 1.64.

## Discussion

The primary aim of this longitudinal study was to examine the existence of heterogeneous latent class trajectories based on the 30 risk and protective items of the SAVRY in a large sample of male juvenile offenders. The identification of risk/need trajectories explores an imperative issue for need-oriented risk management: the capacity of risk/need items to change over time. Research on the changeability of risk/need items of juvenile offenders is scarce but also highly relevant because risk/need assessment tools increasingly inform risk reduction strategies for this population [27]. However, there is very little knowledge about the capacity of these tools to measure change over time and the current study is, as far as we know, the first study to investigate the heterogeneity of trajectories of juvenile offenders on SAVRY risk/need items.

The heterogeneous trajectories that were identified in each of the five risk/need areas of the SAVRY were characterized by the high quality of separation between the trajectories. This indicates that the latent trajectories were distinct from each other. Regarding the principal question of this study it is important to note that the unique patterns of changes in risk/needs of the majority of the identified latent class trajectories showed significant changes over time. In the risk/need area antisocial behaviour, three trajectories were identified, of which two, the mild persisters and the rapid escalators, indicated significant change over time. However, the change in the mild persisters was very minimal at .08 points on a range of 0–1, what would be similar to a 1-point change in SAVRY score (e.g., the change of one risk factor from moderate to high).

Similarly, two of three trajectories of the risk/need area family functioning, the mildly problematic and the increasingly problematic trajectories, showed significant change over time. The change exhibited among juveniles within these two trajectories was unexpected since the items of the family functioning risk/need area are static risk factors. Although unexpected, these results may illustrate that during the adolescent’s contact with the juvenile justice system more (often potentially traumatic) events that occurred within the family context can be identified. Notwithstanding the static nature of these items, they can generate important information [28]; in particular for possible trauma informed interventions [29].

In the area of personality, one of the risk/need areas fully composed of dynamic risk and protective factors, all four trajectories showed significant change over time. However, the highly problematic group improved a very minimal .02 points in risk/needs and the mildly problematic group, the largest class with low risk/needs, showed a very limited increase over time (with .03 points). The trajectory of the rapid responders showed an important improvement of .35 points (which coincides with a 5-point change in SAVRY score) in risk/needs over 18 months, but represented only 5.3% of the sample. By contrast, the trajectory of the rapid escalators (4.3%) accelerated in the opposite direction and increased .35 points in risk/needs over the same 18-month period. These results illustrate the heterogeneity of the change patterns in which different groups of adolescents developed in opposite directions over time during the 18-months study time-frame.

Of the four trajectories of the social support risk/need area, the poorly integrated class was the only one that did not show significant change over time. Furthermore, the increase of .07 points during the 18-months period for the mildly impaired integrators was very limited. Conversely, the trajectory of the rapid integrators had an accelerated reduction in risk/needs (.41 points) during the first 12-months, to increase .09 point in the last 6-months. In comparison, the rapid deterioraters experienced a swift increase in risk/needs of .44 points during the first 9-months, although they did improve somewhat (.15 points) during the remaining 9-months of the trajectory.

In the last risk/need area, treatability, the change over time of the mild resisters (increase of .04 points) and the high resisters (improvement of .07 points) was also minimal. The trajectories of the rapid responders and the rapid escalators illustrated, as the rapid deterioraters and the rapid integrators of the risk/needs area Social support, the possible curvature shape of risk/need trajectories of juvenile offenders. After the baseline assessment the risk/needs trajectory of the rapid escalators group of 4.9% of the sample increased during the first three assessments with .41 points (equivalent to four points on this SAVRY risk/need area), to subsequently decrease with .12 points over the following 9 months. At 13% of the sample, the rapid responders group was the most prevalent class that exhibited a large improvement (.40 points) in risk/needs during the first 9-months; however, this group then slowly increased (.10 points) towards the end of the study period.

In summary, the large classes with high risk/needs did not change over time or showed limited improvement in risk/needs during the 18-month follow-up period. The trajectories with low risk/needs generally remained low and presented a very limited increase in risk/needs. Between 5 and 13% of the juveniles had large reductions in their risk/needs levels; however, approximately 5% of the juveniles had a negative development during the intervention, showing large increases in risk/needs.

Trajectories with extensive change over time in opposite directions were observed in three of the risk/need areas. A visual examination of the graphs in Fig. 2 would suffice to gauge the loss of essential information in all five-risk/need areas if the trajectories were averaged. With the vast majority of research relying on analyses that reflect average sample differences, homogeneous change is assumed and the identification of unique heterogeneous change patterns could be hidden. The heterogeneity of the risk/need trajectories in the current study is consistent with the DLC criminology theories. Similar to the delinquency trajectories [5, 14], the risk/need trajectories of juvenile offenders in the present sample demonstrate that change over time is likely to be heterogeneous.

Our second research question was what characteristics identified from prior research including demographics (age), criminal history (prior violent and non-violent offenses, previously detained), and shifting between sections of the juvenile justice services could differentiate between the risk/needs trajectories. We found that the male juveniles in the low risk/needs trajectories, in comparison with the remainder trajectories, were older in age, and had a low frequency of prior offenses, a lower likelihood of previous detention, shorter current sentences and experienced a very low frequency in sector changes within the juvenile justice system, indicating a smooth transition through the system. The trajectories with high risk/needs and the rapid increasing trajectories were generally the groups of offenders with more prior offenses, longer current sentences and a higher likelihood of multiple sector changes in the juvenile justice system, indicating a problematic transition through the system. The trajectories that presented a large decrease or increase in risk/needs over time were younger in comparison with the high and low risk/need trajectories. However, the decreasing trajectories were generally not very different from the high risk/need and the increasing trajectories. This lack of differences may be explained by the historical nature of the majority of the covariates and that in this study we did not have access to treatment information. Nonetheless, the identified characteristics of the trajectories with low, high and escalating risk/needs are in line with the risk-needs-responsivity (RNR) model [3] in that the most problematic groups were identified with high risk/needs, and the least problematic groups with low risk/needs.

Previous studies have suggested that interventions in juvenile justice services need to be improved and insufficiently target criminogenic needs [7–9]. Unfortunately, in the current study we did not have the data to verify these findings. However, adolescents’ capacity to change over short periods of time has been widely documented [1, 5]. Another possibility is that SAVRY may have detected fever changes than actually occurred. This would suggest that the tool is not sufficiently sensitive to measure changes that juvenile offenders experience during their transition through the juvenile justice system. The results of the present study are consistent with the limited number of studies on the measurement of change with SAVRY assessments [9, 12, 13]. This small body of work suggests that SAVRY has difficulties in measuring change in risk/needs of adolescent offenders. Viljoen and colleagues recently suggested several potential strategies to enhance risk assessment tools’ sensitivity to change [9]. One of their suggestions was a shorter time frame to assess the items. SAVRY uses time frames that refer “to the latest 6-month period” [11] for the dynamic risk items and “the factor has been active or present over the preceding year” regarding the protective items. In assessing future behaviour the large majority of risk assessment tools orientate the time frames to assess the items backwards, in the direction of the history of a person: the past year or the past 3–6 months. But if the objective is the appraisal of the likelihood of future violent or delinquent behaviour of a person, it might be more logical to orient the time frames of the dynamic items towards the coming period, for example the forthcoming 3–6 months. The SAPROF is the only tool with a time frame oriented towards the future [30]. Results of the SAPROF are promising since it is one of the few adult tools that has shown the ability to measure change over time in risk/needs [31].

## Limitations and future directions

Our findings need to be considered in the context of the current study’s limitations. First, the use of assessments performed in a naturalistic setting has clear advantages, but using data from daily clinical practice also presents challenges. Previous studies have reported that the use of assessment measures by professionals in the field can result in lower reliability [32, 33]. However, various field studies have shown that SAVRY assessments are completed with sufficient reliability in juvenile justice field settings [17, 34]. Second, our study did not have access to specific information on the nature and quality of the intervention programs provided for the juveniles, nor the intensity of these interventions. Future studies should attempt to obtain information regarding treatment and management that would provide insight into the extent to which the intensity and/or specific features of these interventions are associated with the various risk/need trajectories. Finally, in the present study we focused on the internal sensitivity to change through the identification of risk/need trajectories of juveniles based on their evolution on risk/need areas as they progressed through the juvenile justice system. The association between risk/needs and offending trajectories could contribute importantly to the insight of different developmental paths of juvenile offenders during their adolescence and their course through juvenile justice services and adjust treatment programs for specific subgroups is an important avenue for future research. That line of research could help determine if juveniles in the risk/need trajectories that show large improvements coincide with ‘desisters’ and the high risk/need groups correspond with life-course persistent offenders, two groups often identified in delinquency trajectories [6].

Risk assessment research has principally been based on cross-sectional research designs and between-individual differences. To validly use risk assessment tools to orient and evaluate risk reduction strategies, more knowledge about the changeability of risk/need items of these tools should become available. Analogue with the DLC theories and the numerous studies on heterogeneous criminal career trajectories, longitudinal research on the development of risk/needs over time could be employed on a broader scale. This type of research could test the heterogeneity of risk/need trajectories, based on items of risk assessment tools and accordingly evaluate their sensitivity to measure within-individual changes over time. It would also be an opportunity to test if future-oriented time frames could improve the sensitivity to measure change over time.

Although SAVRY is a promising tool for risk assessment, recent research suggests it could be insufficiently sensitive to change over time [9, 13]. Since it is one of the most widely used risk assessment tools for adolescents there are large quantities of data available from research and implementation in juvenile justice systems worldwide [35]. It might be worthwhile to explore if this wealth of data, and the valuable experiences of its implementation in juvenile justice systems [36], could be employed to improve SAVRY’s sensibility to change over time. A data driven improvement of SAVRY could also stimulate the field towards more effective prevention and risk management.

## Conclusions

In line with the developmental and life-course criminology theories this study illustrated that latent class trajectories on the basis of SAVRY risk and protective items can be heterogeneous and indicate distinct rates of change over time. Several of these trajectories revealed different patterns of changes in opposite directions within the same risk/need area, demonstrating the importance of examining change in a manner that does not lose sight of this heterogeneity. Although SAVRY is a promising tool for risk assessment, this study, taken into the light of recent research [9, 13] suggests it could be insufficiently sensitive to change over time.
